# Distribution of Saponins in the Sea Cucumber *Holothuria lessoni*; the Body Wall Versus the Viscera, and Their Biological Activities

**DOI:** 10.3390/md16110423

**Published:** 2018-11-01

**Authors:** Yadollah Bahrami, Wei Zhang, Christopher M. M. Franco

**Affiliations:** 1 Medical Biotechnology, School of Medicine, College of Medicine and Public Health, Flinders University, Adelaide, SA 5042, Australia; wei.zhang@flinders.edu.au; 2 Pharmaceutical Sciences Research Center, Kermanshah University of Medical Sciences, Kermanshah 6714415185, Iran; 3 Medical Biotechnology, Faculty of Medicine, Kermanshah University of Medical Sciences, Kermanshah 6714415185, Iran; 4 Centre for Marine Bioproducts Development, College of Medicine and Public Health, Flinders University, Adelaide, SA 5042, Australia

**Keywords:** triterpene glycosides, saponin, sea cucumber, mass spectrometry, MALDI, ESI, LC-MS, Holothuroidea, marine ginseng, structure elucidation, marine invertebrate, natural products, bioactive compounds, antifungal, antibacterial, antioxidant

## Abstract

Sea cucumbers are an important ingredient of traditional folk medicine in many Asian countries, which are well-known for their medicinal, nutraceutical, and food values due to producing an impressive range of distinctive natural bioactive compounds. Triterpene glycosides are the most abundant and prime secondary metabolites reported in this species. They possess numerous biological activities ranging from anti-tumour, wound healing, hypolipidemia, pain relieving, the improvement of nonalcoholic fatty livers, anti-hyperuricemia, the induction of bone marrow hematopoiesis, anti-hypertension, and cosmetics and anti-ageing properties. This study was designed to purify and elucidate the structure of saponin contents of the body wall of sea cucumber *Holothuria* *lessoni* and to compare the distribution of saponins of the body wall with that of the viscera. The body wall was extracted with 70% ethanol, and purified by a liquid-liquid partition chromatography, followed by isobutanol extraction. A high-performance centrifugal partition chromatography (HPCPC) was conducted on the saponin-enriched mixture to obtain saponins with a high purity. The resultant purified saponins were analyzed using MALDI-MS/MS and ESI-MS/MS. The integrated and hyphenated MS and HPCPC analyses revealed the presence of 89 saponin congeners, including 35 new and 54 known saponins, in the body wall in which the majority of glycosides are of the holostane type. As a result, and in conjunction with existing literature, the structure of four novel acetylated saponins, namely lessoniosides H, I, J, and K were characterized. The identified triterpene glycosides showed potent antifungal activities against tested fungi, but had no antibacterial effects on the bacterium *Staphylococcus aureus*. The presence of a wide range of saponins with potential applications is promising for cosmeceutical, medicinal, and pharmaceutical products to improve human health.

## 1. Introduction

Sea cucumbers are known as slow-moving invertebrates, in which most species are nocturnal and benthic. They vary in size, shape, colour, and flavours. They have different pharmacological, nutraceutical, and medicinal activities due to the remarkable differences in the type and quantity of saponins, as well as the biodiversity of their species. These differences might also result from the localisation of saponins. Sea cucumbers are referred to as “marine ginseng” since they are a prolific source of bioactive compounds with many functions and are a potential source of biomedical and agrochemical products to treat or prevent many diseases.

*Holothuria lessoni*, commonly known as golden sandfish, belongs to the family Holothuriidae, class Holothuroidea, order Aspidochirotida, phylum Echinodermata. The colouration of this relatively new-identified holothurian is highly variable from dark greyish black to beige with black blotches and spots or beige without black spots [1,2]. Sea cucumbers are a delicacy in Chinese cuisine. This species is among the species with the highest demand for luxury seafood in Asia [3], which contains a high diversity of saponins in the viscera with a potential medicinal value. Purcell [3] also stated that *H. lessoni* and *H. scabra* are the most valuable tropical holothurians in dried (beche-de-mer) seafood markets in China. The processed (dried) *H. lessoni* is marketed in Hong Kong in retail markets with prices ranging from USD 242 to 787 per kg [1].

Holothurians, commonly known as sea cucumbers, generate a wide range of distinctive biologically and pharmacologically important compounds including triterpene glycosides, fatty acids, minerals, carotenoids, sphingosine, bioactive proteins (collagen, gelatine, peptides, amino acids), vitamins, mucopolysaccharides, glycosaminoglycan (chondroitin/fucan sulphates), fucoidan, phenolic, and flavonoids [4,5]. The presence and power of these active ingredients have led to a rapid growth and development in various biomedical and functional food industries, important to human health.

Sea cucumbers are a potential source of high-value-added substances with therapeutic applications in nutraceutical, cosmeceutical, medicinal and pharmaceutical products. Sea cucumber is consumed as traditional folk medicine in many Asian countries to cure diseases like rheumatoid arthritis, joint pain, tendonitis, osteoarthritis, cardiovascular, ankylosing spondylitis, arthralgia, tumours, fungal infection, gastric, impotence, frequent urination and kidney deficiency, high blood pressure and muscular disorders [6]. Thereby, the medicinal and beneficial influences of functional sea cucumbers on human health have been validated through scientific literature and have exhibited therapeutic value such as controlling excessive cholesterol levels, wound healing, neuroprotective, antimicrobial, anti-malaria, antithrombotic, anticoagulant, antioxidant, and anti-ageing (anti-melanogenic and anti-wrinkle) [4]. Many studies revealed that the health benefits and therapeutic properties of sea cucumbers are due to the presence of triterpene glycosides (saponins).

Saponins are water-soluble constituents. Among the marine organisms, triterpene glycosides (saponins) are predominantly identified in sea cucumber [7], starfish [8] and sponges. The chemical structures of saponins produced by sea cucumbers are unique and vary remarkably from those of terrestrial saponins. Triterpene glycosides, labelled as the most abundant glycosylated secondary metabolites in sea cucumbers, comprise of a carbohydrate moiety and an aglycone. The aglycone part of marine saponins is either triterpene (C30, sea cucumber) or steroid (C27, starfish). Triterpene molecules are assembled from six isoprene units containing 30 carbon atoms. Their aglycone possesses a molecular weight ranging from 400 to 1000 Da. Over 700 triterpene glycosides have been reported from various species of holothurians with a wide spectrum of chemical structures including sulfated, non-sulfated, and acetylated triterpene glycosides [7]. This diversity highlights their potential functions and commercial applications. Besides, the chemical diversity of saponins makes them more favourable as lead compounds for novel drug discovery.

Sea cucumber saponins are usually triterpene glycosides containing a holostane structure. The aglycone part of these glycosides are mainly derived from a tetracyclic triterpene lanosterol and possess a skeleton of a hypothetical lanostan-3-β-ol-(18-20)-lactone called as holostanol in that the D-ring contains a γ-18(20)-lactone. Besides a number of triterpene glycosides possessing aglycones with 18(16)-lactone or without a lactone ring are also reported [6,7]. Typically, their triterpene glycosides contain a polycyclic nucleus with 7(8)- or 9(11)-double bond, and oxygen-bearing substituents are prominently linked to C-12, C-17 or C-16. The lateral chain of aglycones may also contain different substituents namely hydroxy or acetate group, which can further enhance the diversity of saponins.

Their oligosaccharide moieties consist of up to six monosaccharide units, linked exclusively to the C-3 of the aglycone. The sugar residues mainly compose of d-xylose (Xyl, X), d-quinovose (Qui, Q), 3-*O*-methyl-d-glucose (MeGlc, MG), 3-*O*-methyl-d-xylose (MeXyl, MX) and d-glucose (Glc, G), and sometimes 3-*O*-methyl-d-quinovose (MeQui, MQ), 3-*O*-methyl-d-glucuronic acid (MeGlcA) and 6-*O*-acetyl-d-glucose (AcGlc). The molecular weight of prominent sugar residues are as hexose (162 Da), methylpentose or deoxyhexoses (146 Da), and pentose (132 Da) and methylhexose (176 Da). In the oligosaccharide chain, the first monosaccharide unit is always a Xyl, whereas the methylated monosaccharides, namely, MeGlc and/or MeXyl and/or MeQui are always the terminal sugars. 

Saponins are widely distributed in sea cucumber species. In recent decades, these natural metabolites have gained great attention worldwide due to their unique features: rich sources, low toxicity, and high efficiency with few side effects [9]. Triterpene glycosides of sea cucumber are known to possess a broad range of medicinal and physiological activities [10,11]. The medical potency of sea cucumber saponins exhibits plentiful health benefits due to their cardiovascular, ant-diabetic, hypoglycaemia, anti-oxidant, anti-asthma, anti-eczema, anti-inflammatory, anti-arthritic, anti-diabetics, cholesterol-lowering effect, immunomodulator, cytotoxic, anti-parasitic, anti-viral, antifungal [7,12], anticancer [13,14], anti-angiogenesis, anti-proliferative [15], and anti-dementia activities [2]. According to the literature, saponins also possess neuroprotective effects on the diminution of central nervous system disorders, namely Alzheimer’s disease, Huntington’s disease, Parkinson’s disease, and strokes [16]. Saponins are also able to stimulate apoptosis and prevent the growth of tumour cells [7]. Besides, sea cucumber saponins are also reported to have biological activities including lowering hyperlipidemia, regulating fat accumulation, restraining fatty liver, relieving hyperuricemia, controlling blood sugar, inhibiting gout and stimulating the hematopoietic function of bone marrow [9]. Various analytical techniques have been applied to study the structure of saponins.

Nuclear magnetic resonance (NMR) spectroscopy can provide extensive structural information for saponins, but high-quantities of high-purity samples are generally required. Saponins are often extracted as a complex mixture, needing a sequence of purification methods to fulfil the requirements for NMR analysis due to the relatively low concentration of saponins. Applying an NMR for analysing saponins in complex mixture generates signals for the most prominent metabolites, whereas signals of the low content metabolites remind either undetected or largely buried by dominant metabolites. In addition to the sample’s complexity, the weak S/N ratio of NMR signals makes the structure elucidation of saponins very challenging. However, various mass spectrometry (MS) approaches have been documented to be rapid, reliable, sensitive and accurate for the direct analysis of saponins, both in terms of composition and relative proportion. Recently, Decroo et al. reported the successful application of ion mobility mass spectrometry for the analysis of saponins from different sources [17].

The combination of various MS-based approaches, such as matrix-assisted laser desorption/ionization mass spectrometry (MALDI-MS)/MS and electrospray ionization mass spectrometry (ESI-MS)/MS, affords a wealth of structural data on the saponin congeners, without applying sequential purifications. However, the structural determination of compounds is highly reliant on low kinetic energy collision-induced dissociation (CID) which cannot provide a comprehensive structure elucidation in terms of stereochemistry in some cases [18]. Accordingly, in this study, the integration of the counter-current chromatography and mass spectrometry techniques were utilised to purify and deduce the structure of saponins. We believe that it is a powerful and efficient technique for data interpretation of saponin congeners to tackle the structural complexity of saponin congeners. It can also differentiate the structure of isomeric compounds as they generate different MS/MS fingerprint patterns. It is notable that the mass transition of 132 Da, 146 Da, 162 Da, and 176 Da are due to the losses of Xyl (132), Qui (146), Glc (162 Da), and MeGlc (176), respectively. Usually, the simultaneous loss of two sugar units is also observed.

Previously, we thoroughly described the isolation and structure elucidation of a number of saponins in the viscera of *H. lessoni*. This study aims to purify and characterize the saponin congeners in the body wall of *H. lessoni.* This manuscript is the first to describe the distribution of saponins in the body wall of *H. lessoni*. In addition to their biological properties, it addresses the purification and structure elucidation of several holostane glycosides, including many new saponins along with multiple known compounds from the body wall of this species using the same methods as described previously [2,6,11], unless otherwise stated. Due to their structural diversity and amphiphilic nature, saponins provide a potent platform for pharmaceutical, medicinal, cosmeceutical, nutraceutical, and functional food applications.

## 2. Results

Despite the advanced developments in the extraction and purification methods, the isolation and identification of saponins in complex extracts remain challenging due to their similar physico-chemical and amphiphilic properties. We previously reported the isolation and purification of a number of saponins from the viscera of a sea cucumber species, *H. lessoni*, using standard chromatography and high-performance centrifugal partition chromatography (HPCPC) to overcome this issue [2,6,11]. The saponin constituents of the body wall of *H. lessoni* were also investigated using the same protocol to compare the saponin profiles and distribution of saponin congeners within these organs.

### 2.1. HPCPC Purification

**Figure 1:**
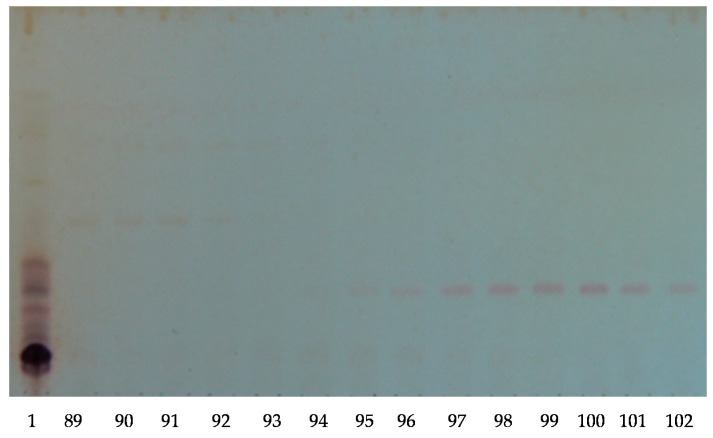
The thin-layer chromatography (TLC) pattern of the high-performance centrifugal partition chromatography (HPCPC) fractions from the purified extracts of the body wall of the *H. lessoni* sea cucumber using the lower phase of the CHCl_3_–MeOH–H_2_O (7:13:8) system. The numbers under each lane indicate the fraction number in the fraction collector. The Fractions 89 to 102 of one analysis (of 130 fractions) are shown. Lane 1 is the saponin enriched iso-butanol extract.

One hundred and forty milligrams of the saponin-rich butanolic extract was fractionated by HPCPC in the ascending mode, and 130 fractions were collected and monitored by TLC as described previously [2,6,11]. The TLC profile of the saponin-enriched sample showed the presence of several bands Figure 1 (lane 1), whereas the TLC pattern of HPCPC fractions exhibited the existence of one band in the majority of fractions (Figure 1). Conducting HPCPC is critical for the separation of isomeric saponins. As a typical example, the TLC profile of HPCPC Fractions 89–102 is shown in Figure 1.

### 2.2. Mass Spectrometry Analysis of Saponins

The chemical profile of saponins was assigned by mass spectrometry using combinations of MALD-MS/(MS) and ESI-MS/(MS) in the positive and/or negative ion mode(s).

#### MALDI-MS and ESI-MS Analyses of Saponins from the Body Wall of *H. lessoni*

Saponin HPCPC fractions from the body wall of *H. lessoni* were analysed by MALDI-MS and ESI-MS, and MS/MS as described in detail previously [2,6,11]. The mass spectra were recorded within a *m*/*z* mass range of 400–2200 Da. The MALDI-MS and MS/MS were performed in the positive ion mode, while ESI-MS and MS/MS were conducted in both positive and negative ion modes. The observed ions clearly all correspond to ionized saponins. All detected ions in the positive ion mode were sodium-coordinated species such as [M − H + 2Na]^+^ and [M + Na]^+^ corresponding to sulphated and non-sulphated saponins, respectively. We have actually conducted a comprehensive literature review on the structure of saponins analysed by MS, and built an extensive MS library data to develop a stepwise protocol for the interpretation of MS spectra. The first step was performed to obtain the mass-to-charge ratio of all saponin ions and define the elemental composition of the corresponding saponin contents and their molecular weights. However, in the second step, MS/MS was applied to elucidate the structure of saponin ions by which ions of interest were mass-selected and subjected to CID, resulting in fragmented ions. The mass transition between the fragmented ion peaks is critical for reconstructing the structure of the parent ions.

MALDI and ESI-MS intensities were used to compare saponin compositions within each organ. Besides, they were used to estimate the relative proportion of saponin congeners in the extracts. More than 89 saponin congeners were found in the body wall of sea cucumber *H. lessoni*, which are summarised in Table 1. Around 80 saponins were common between the body wall and the viscera. Nine saponin congeners were found solely in the body wall as compared to the viscera (Table 1).

**Figure 2:**
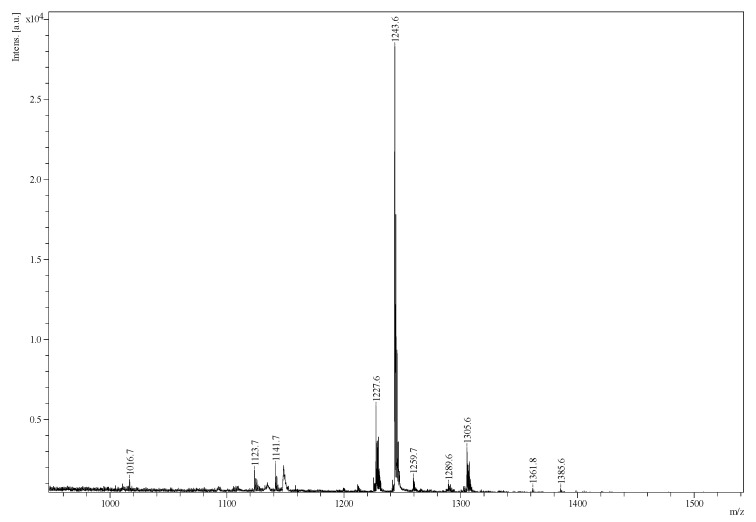
The matrix-assisted laser desorption/ionization mass spectrometry (MALDI-MS) fingerprint of saponin enriched iso-butanol extract over the mass range of 950–1550 *m*/*z* from the body wall of *H. lessoni*.

Twenty-three major saponin peaks were detected at *m*/*z* 905.4, 1069.5, 1071.5, 1087.5, 1107.5, 1109.5, 1123.5, 1125.5, 1141.5, 1199.5, 1211.5, 1227.5, 1229.5, 1243.5, 1287.6, 1289.6, 1303.6, 1305.6, 1361.7, 1461.7, 1463.7, 1475.7, and 1477.7 in the body wall of *H. lessoni* (Figure 2). These intense peaks could each correspond to at least one triterpene saponin congener. Compounds were assigned on the bases of the *m*/*z* values, isotope distributions, and fragmentation patterns.

The most abundant saponin peaks were detected at *m*/*z* 1141.5, 1227.5, 1229.5, and 1243.5, which corresponded to Desholothurin A (Nobiliside 2a) (*m*/*z* 1141.5) [28,29], Fuscocinerosides B or C (*m*/*z* 1227.5)—which are isomers [2,28,37]—Holothurin A_2_ (*m*/*z* 1229.5) [43], and Holothurin A (*m*/*z* 1243.5) [11,28,38,39,47,61], respectively. These abundant saponin congeners were sulphated triterpene glycosides (Table 1) except for the ions monitored at *m*/*z* 1141.7. Likewise, in the viscera, the most predominant peak at *m*/*z* 1243.5 corresponded to Holothurin A, which was followed by the ions at *m*/*z* 1227.5, 1229.5, 1305.6, and 1141.7. However, in the viscera, the ions at *m*/*z* 1243.5, 1141.5, 1305.6, 1259.5, and 1227.5 were from the five most intense saponins. In all the sulphated saponins ranging from *m*/*z* 900 to 1400, it was xylose that was sulphated.

The distribution of saponin in the cuvierian tubules and body wall of *H. forskali*, in the same family as *H. lessoni*, was investigated using both conventional MALDI and MALDI mass spectrometric imageing (MALDI-MSI) analyses [30,55]. This group reported eight major intense peaks at *m*/*z* 1125, 1141, 1287, 1303, 1433, 1449, 1463, and 1479. All of these glycosides were defined as non-sulphated saponins, while the major abundant saponins in the *H. lessoni* were sulphated congeners (except the ions at *m*/*z* 1141.5).

**Figure 3:**
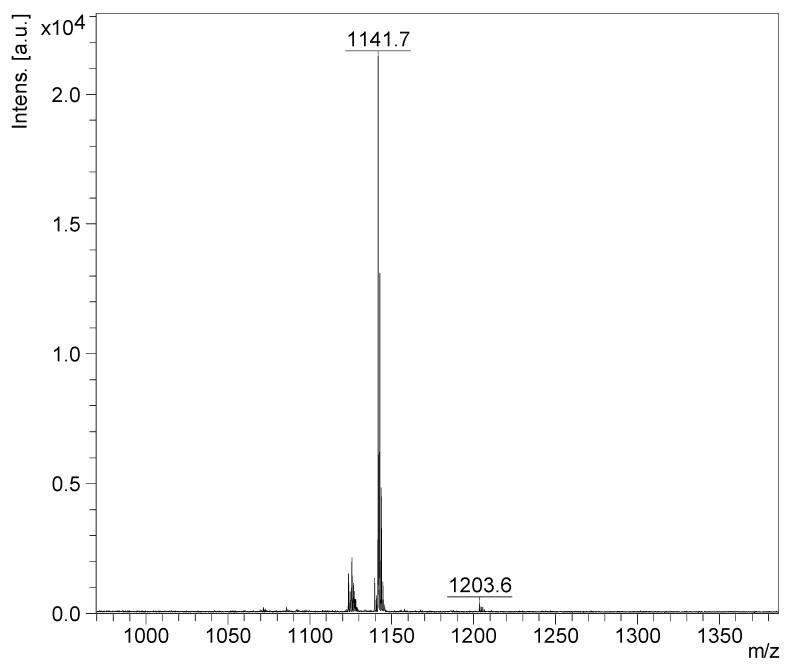
The MALDI-MS fingerprint of Fraction 110. The major peak at *m*/*z* 1141.7 corresponded to Desholothurin A.

HPCPC fractions were also analysed. For instance, the positive ion mode MALDI-MS of Fraction 110 over a mass range of 950–1400 *m*/*z* is shown in Figure 3. This spectrum illustrates the presence of one major peak at *m*/*z* 1141.7 corresponding to Desholothurin A [29].

**Figure 4:**
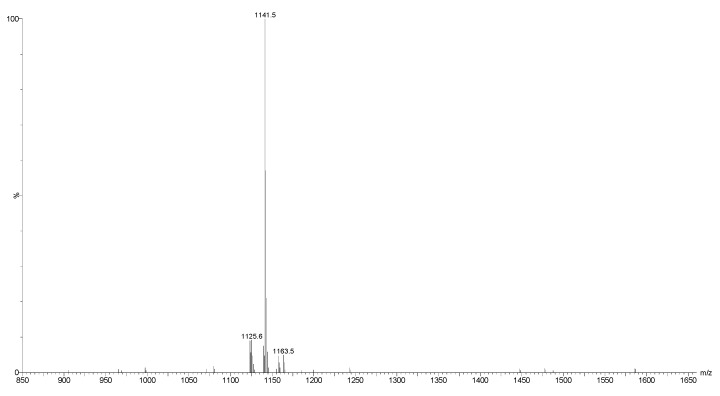
The electrospray ionization mass spectrometry (ESI-MS) spectrum of Fraction 110. The major peaks corresponded to Desholothurin A.

Both positive and negative ion modes ESI-MS were also performed on the fractions. As an example, the positive ion mode ESI-MS spectrum of Fraction 110 is shown in Figure 4. This spectrum indicated the presence of the major ions at *m*/*z* 1141.5, corresponding to Desholothurin A. Therefore, the MALDI-MS data was corroborated by ESI-MS analysis.

**Figure 5:**
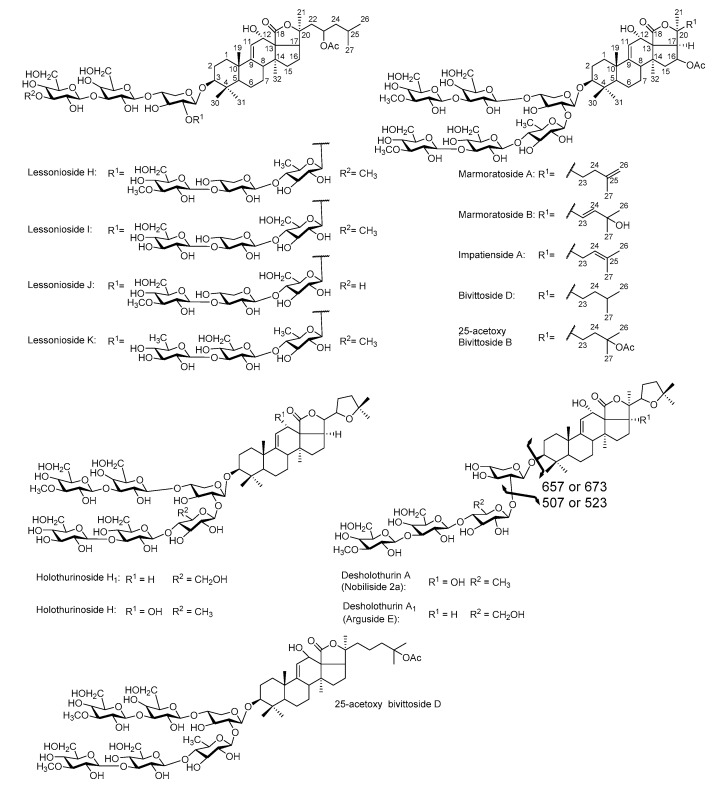
The structures of some of the newly identified saponins from the body wall of *H. lessoni*, as representative.

A chemical analysis by MALDI- and ESI-MS/MS of the HPCPC fractions identified several novel along with multiple known saponins. The molecular structures of some of the identified compounds are illustrated in Figure 5. The isobutanol and HPCPC fractionated samples indicated 26 sulphated and 63 non-sulphated saponin ions.

### 2.3. Saponin Profiles by Negative-Ion ESI-MS

**Figure 6:**
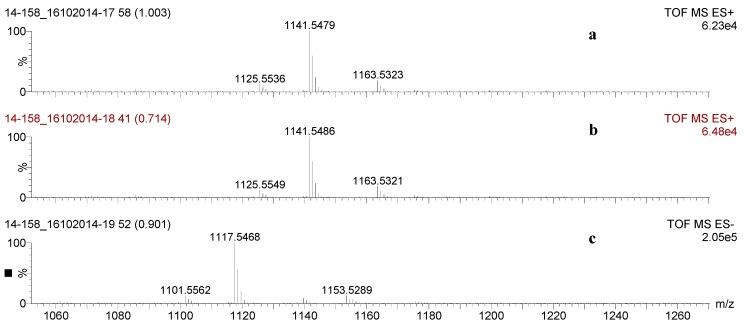
The saponin profile of Fraction 110 by ESI-MS in both the positive (**a**,**b**) and negative (**c**) ion modes. The 24 u or Da mass discrepancy between the positive and negative ion modes for an individual ion indicates the compound is non-sulphated saponin.

The result of the positive ion mode was validated by the negative ion mode under conditions similar to those used for the positive ion mode. The analysis of saponins in the negative ion mode facilitated the calculation of the molecular formula of compounds as it showed the presence of the number of Na ions in the molecules, and also the presence or absence of sulphate groups. For instance, the ions detected in both the positive and negative ion modes ESI-MS of HPCPC Fraction 110 are displayed in Figure 6, which demonstrated ions detected in both positive [M + Na]^+^ (Figure 6a,b) and negative [M − H]^−^ (Figure 6c) ion modes between 1050 and 1275 Da. Three main peaks at *m*/*z* 1125, 1141, and 1163 in ESI-MS^+^ generated peaks at *m*/*z* 1101, 1117, and 1139 in the negative ion mode [M − H − Na]^−^ ESI-MS, respectively, indicating the presence of only one Na atom in their chemical formulae. The analysis of saponins in the negative ion mode involves the loss of a proton. As can be noted from the spectra, the mass discrepancy between the positive and negative ion modes for an individual ion was 24 u or Da, representing the loss of a sodium atom and a proton, and showing that there was no sulphur present. Therefore, the mass discrepancy between the sodiated saponins and the deprotonated saponins is 24 u. However, in the case of a sulphated saponin, the mass discrepancy between these two modes of ionisation was 46 u, showing the presence of two Na atoms which implies the presence of sulphur in the molecule.

### 2.4. Structure Elucidation of Saponins by Tandem Mass Spectrometry Analysis

The appropriate HPCPC fractions were pooled on the basis of their similar Rf values on TLC and concentrated to dryness. The saponin content of each HPCPC fraction was then profiled by MALDI-MS, ESI-MS, and -MS^2^. Tandem mass spectrometry analysis (MALDI and ESI) afforded crucial information about the chemical structure and elemental composition of individual saponins. Isomeric saponins were also differentiated following HPCPC purification [2,11,62]. However, in some cases, the definitive structure elucidation of saponins requires NMR analysis. It is notable that the low kinetic energy CID used here had no fragment in the core of the aglycone, whereas the side chain of the aglycone was cleaved in some cases, which was consistent with observations by Demeyer, et al. [63]. To describe the procedure, the tandem mass spectrometry analysis of a few saponin ions will be discussed.

Our previous MS^2^ analyses of saponins revealed the key diagnostic ion peaks, namely the main fragmentation ions, generated by the cleavage of the glycosidic bonds, yielding oligosaccharide and monosaccharide fragments [2,6,11]. These characteristic peaks and unique fragmentation pattern provide vital structural information about the MW of the aglycones, the glycoside linkage, nature, number, sequence, and type of monosaccharaide units in the carbohydrate moiety, as well as the presence or absence of different groups such as acetoxy and/or sulphated moieties and their positions. Besides, other visible peaks originated from the cleavage of the lateral chain of aglycone and the loss of other neutral molecules, including H_2_O and CO_2_. In some cases, we observed the simultaneous loss of two sugar units.

Collisional induced-dissociation can also cleave the lateral chain of the aglycone and generate a wealth of information about the structure of the nucleus and side chain. For instance, the typical mass transitions of 60 and 104 u from the parent ions correspond to the losses of acetoxy group (acetic acid, C_2_H_4_O_2_) and [C_2_H_4_O_2_ + CO_2_] in the aglycone of acetylated triterpene glycosides, respectively. The latter one is a characteristic feature of compounds having an acetoxy group and an 18(20)-lactone moiety. The presence of ion peaks at *m*/*z* 230.15 and 204.13 in the spectrum of triterpene glycosides corresponding to the losses of [C_12_H_22_O_4_] and [C_10_H_20_O_4_] are the common characteristic fragments of saponins with a saturated lateral chain. The side chain fragmentation with 23-oxo substitution led to losses of 100 Da, due to the low energy McLafferty rearrangement of 6-member transition states, which generates the neutral molecule C_6_H_12_O (4-methylpent-1-en-2-ol). Having knowledge of these fragmentation ions enable us to elucidate the structure of novel aglycones.

### 2.5. Structural Determination of Saponins by MALDI MS/MS

**Figure 7:**
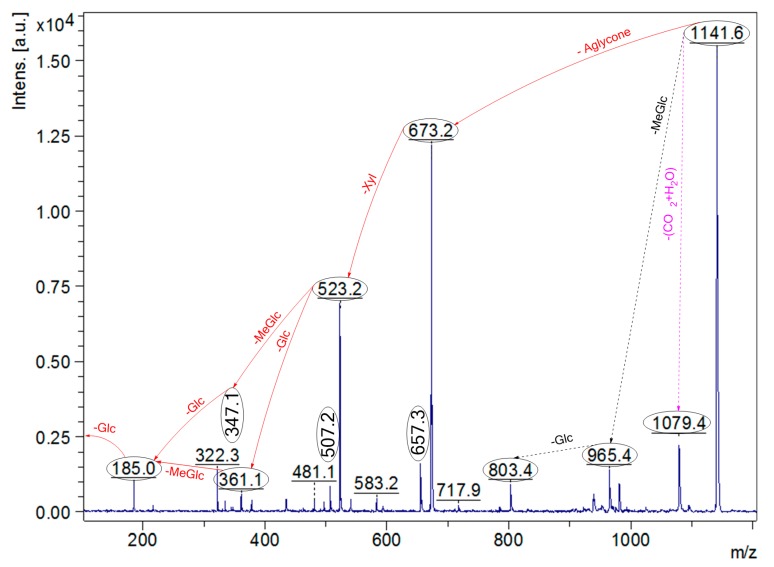
The MALDI-MS/MS profile of the ions at *m*/*z* 1141 from Fraction 55 corresponding to desholothurin A_1_. The sequential losses of aglycone (Agl), Xyl, MeGlc, Glc, and Glc residues yielded the product ions at *m*/*z* 673, 523, 347, and 185, respectively. However, the ion peaks at *m*/*z* 507 and 657 corresponded to the sodiated key diagnostic peak [MeGlc-Glc-Qui + Na]^+^, and the entire sodiated hydrated sugar residues [MeGlc-Glc-Qui-Xyl + H_2_O + Na]^+^ of desholothurin A, respectively.

To validate the structure of saponins, tandem mass spectrometry was conducted on the detected ions. As a typical example, the MALDI-MS/MS profile of the ions at *m*/*z* 1141 from Fraction 55 is shown in Figure 7. The chemical analysis of this ion revealed the structure of desholothurin A_1_ [34]. This conclusion was established by fragment ion peaks at *m*/*z* 673, 523, 361, and 185 in the positive ion mode MALDI-MS^2^, corresponding to the sequential losses of aglycone, Xyl, Glc, MeGlc, and Glc residues, respectively.

#### 2.5.1. Chemical Analysis of Saponins by ESI-MS/MS

**Figure 8:**
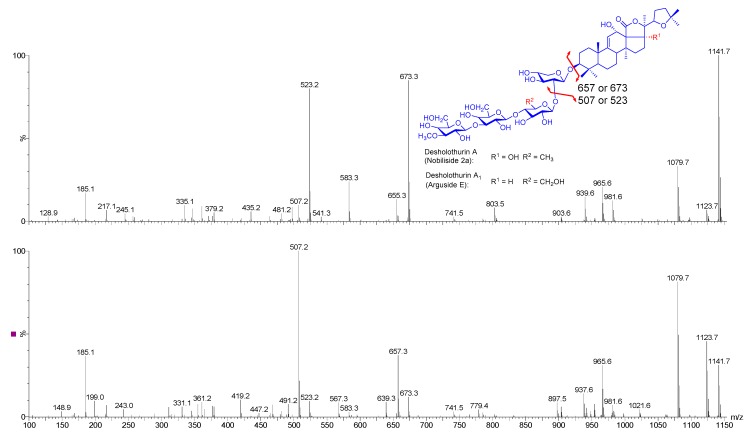
(+) The ESI-MS/MS spectra of the ions at *m*/*z* 1141.7 in Fractions 55 (top) and 110 (bottom). The figure indicates the presence of isomeric compounds. The key diagnostic peak at *m*/*z* 523 corresponding to [MeGlc-Glc-Glc + Na]^+^ revealed the structure of desholothurin A_1_, while the key diagnostic peak at *m*/*z* 507 corresponding to [MeGlc-Glc-Qui + Na]^+^ indicted the structure of esholothurin A. The peak at *m*/*z* 481.2 corresponds to [Glc-Qui-Xyl + Na + H_2_O].

The effective capability of HPCPC in purifying saponins and isomeric saponins was described previously [6,11]. The separation of ions detected at *m*/*z* 1141 is exemplified in Figure 8.

The positive ion mode ESI-MS^2^ spectra of the ions detected at *m*/*z* 1141 from the Fractions 55 (the top spectrum) and 110 (the bottom spectrum) are shown in Figure 8 as representative. These ions corresponded to desholothurin A_1_ (arguside E) and desholothurin A (nobiliside 2a), respectively, which were different in both aglycone and sugar moieties from each other [2,31]. The presence of *m*/*z* 507 and/or 523 ions as the key fragment ions were observed in the MS^2^ spectra of these compounds.

These isomeric compounds showed different MS/MS spectra. The major peak at *m*/*z* 523 (the top spectrum, Figure 8) corresponded to the sodiated key diagnostic peak [MeGlc-Glc-Glc + Na]^+^, and the peak at *m*/*z* 673 generated by the loss of the Agl moiety corresponded to the entire sodiated hydrated sugar residue [MeGlc-Glc-Glc-Xyl + Na]^+^. Therefore, this compound had an aglycone with a molecular weight of 468 Da. Our analysis inferred a tetraoside structure for these ions. This analysis revealed the structure of tetrasaccharide triterpene glycoside, corresponding to Desholothurin A_1_.

The prominent peaks at *m*/*z* 507 and 657 (the bottom spectrum, Figure 8) corresponded to the sodiated key diagnostic peak [MeGlc-Glc-Qui + Na]^+^, and the entire sodiated hydrated sugar residues [MeGlc-Glc-Qui-Xyl + Na]^+^, respectively. The latter ion indicated that this compound had an aglycone with a molecular weight of 484 Da. The consecutive losses of the aglycone, Xyl, Qui, and Glc residues followed the MeGlc afford product ions at *m*/*z* 657.3, 507.2, 361.2, and 199.0. These findings revealed the structure of this compound as desholothurin A. Therefore, the analysis of data showed that HPCPC could separate the isomeric congeners in some cases. The integration of the counter-current chromatography and mass spectrometry techniques was an efficient and reliable approach for the purification and structure elucidation of saponins.

**Figure 9:**
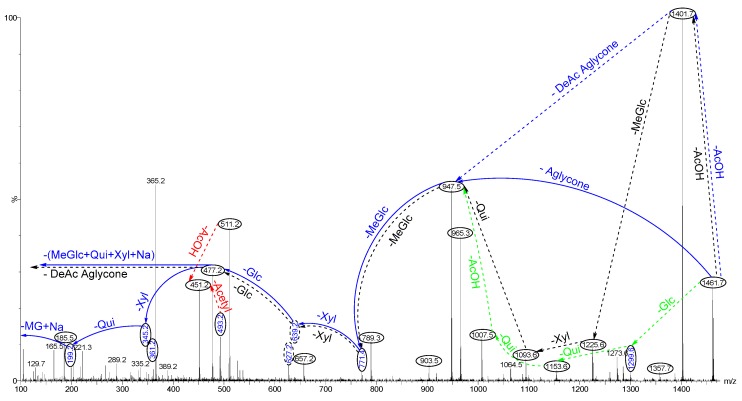
The positive ion mode ESI-MS/MS spectrum of ions detected at *m*/*z* 1461.7 from Fraction 95. The spectrum reveals the presence of different aglycones and sugar residues in the isomeric saponins. The full and dotted arrows demonstrate the three main feasible fragmentation pathways. The fragmentation pattern of ions at *m*/*z* 1461.7 reveals the structure of acetylated saponins lessoniosides H and K as a representative. The blue arrows show the decomposition of the isomeric congeners Lessonioside H, whereas the green arrows indicate the fragmentation patterns of lessonioside K. The loss of the acetoxy group from the ions at *m*/*z* 511.2 generates ions at *m*/*z* 451.2, which corresponds to hydrated three sugar units [Xyl-Xyl-MeXyl + H_2_O + Na].

As an example, the positive ion mode ESI-MS/MS for the ions detected at *m*/*z* 1461 [M + Na]^+^ from Fraction 95 is shown in Figure 9. These ions displayed an *m*/*z* value of 1437 [M − H]^−^ in the negative ion mode ESI-MS, indicating that there was no sulphur group in the molecular structure.

CID triggers three feasible independent fragmentation pathways of cationised parent ions shown in full and dotted arrows (for more details please refer to References [2,5,6,7,11]). The successive losses of the acetic acid (AcOH), deacetylated aglycone (DeAc Agl), 3-*O*-methyl-d-glucose (MeGlc), d-xylose (Xyl), d-glucose (Glc), Xyl, and d-quinovose (Qui) residues (blue arrows) were followed by MeGlc yielded ion fragments at *m*/*z* 1401.7, 947.5, 771.4, 639.2, 477.2, 345.2, and 199.2, respectively, in one of the new isomers for which we propose the name lessonioside H. Further, the sequential losses of MeGlc, Xyl, Qui, acetyl group, MeGlc, Xyl, Glc, and the deacetylated aglycone from the parent ions generated the fragment ions at *m*/*z* 1285.6, 1153.6, 1007.5, 965.3, 789.2, 657.2, and 477.2, respectively. This sequence of fragmentation confirms the structure of the new saponin, lessonioside H. As Figure 9 illustrates this triterpene glycoside contains the ion at *m*/*z* 493.2, corresponding to the key diagnostic sugar residue [MeGlc-Glc-Xyl + Na]^+^. The black dotted arrows also corroborated the structure of lessonioside H. Alternatively, the consecutive losses of the deacetylated aglycone and acetic acid (AcOH) followed by sugar residues yielded ion fragments at *m*/*z* 1007.5 and 947.5, respectively. The latter ion corresponded to the sodiated sugar moiety generated by the loss of the Agl. This sequence of fragmentation confirmed the presence of an acetoxy group. The green dotted arrows indicate the decomposition patterns of lessonioside K, a new acetylated triterpene glycoside.

One of the new isomers was found to be identical with intercedenside A (C_55_H_83_NaO_25_S), a sulfo-acetylated saponin was isolated from *Mensamaria intercedens* sea cucumber [36]. The MS^2^ analyses of ions at *m*/*z* 1461.7 revealed a similar fingerprint profile with those reported for lessoniosides, which were isolated and characterised from the viscera of this species, in particular with Lessonioside A where the signals were coincident [6]. In addition, the sugar moiety of this novel isomeric compound was found to be identical to those of lessonioside A, confirming the constituents of the hexasaccharide chain. This novel triterpene glycoside had a holostane aglycone containing an 18(20)-lactone with a 9(11)-double bond and acetoxy group at C-23. We named these isomeric compounds lessoniosides H, I, J, and K.

Further, these isomers differed from holothurinoside H (marmoratoside B) in the sugar moieties. holothurinoside H generates a peak at *m*/*z* 507 corresponding to MeGlc-Glc-Qui under a positive ion mode mass spectrometry [30]. However, no peak was detected at *m*/*z* 507 corresponding to the key diagnostic ion [MeGlc-Glc-Qui + Na]^+^ from the ions at *m*/*z* 1461.

Moreover, Sun, et al. [64] reported a lanostane-type triterpene glycoside, impatienside A, with a molecular weight [M + Na]^+^ of 1447 (C_67_H_108_O_32_), which had a peak at *m*/*z* 1423 [M − H]^−^ in the negative ESI-MS, isolated from the sea cucumber *Holothuria impatiens*, and contained a double bond at the C24 position (ions 507 and 493), along with a structurally related known compound, bivittoside D [M + Na]^+^ 1449 (C_67_H_110_O_32_) and by negative ESI-MS *m*/*z* 1425 [M − H]^−^, similar to impatienside A, without a double bond. However, Yuan, et al. [59] described a structure with a double bond at the position of C25 instead of C24 for this compound. This compound was detected in both the viscera and body wall of *H. lessoni*. However, it was found to be more intense in the body wall than the viscera.

Yuan et al. [59] isolated several saponins including marmoratoside A [M + Na]^+^ 1447 (C_67_H_108_O_32_), 17α-hydroxy impatienside A [M + Na]^+^ 1463 (C_67_H_108_O_33_), marmoratoside B [M + Na]^+^ 1463 (C_67_H_108_O_33_), 25-acetoxy bivittoside D [M + Na]^+^ 1507 (C_69_H_112_O_34_), together with known glycosides impatienside A and bivittoside D from the sea cucumber *B. marmorata.* These compounds were also identified in *H. lessoni*.

Our analysis revealed the presence of an ion peak at *m*/*z* 1435 [M + Na]^+^ in the positive ion mode MS which showed a signal at *m*/*z* 1411 [M − H]^−^ in the negative-ion mode ESI-MS. Tandem mass spectrometry revealed the isomeric structure of the ions at *m*/*z* 1435. The assignment of fragments revealed that these ions were isomeric compounds. These saponins were also common between the body wall and viscera. Wang, et al. [65] reported variegatuside D with a chemical formula C_59_H_96_O_27_ at *m*/*z* 1259 [M + Na]^+^, which might be produced by loss of MeGlc from the ions at *m*/*z* 1435.

Another novel isomeric saponin ion detected at *m*/*z* 1221.5 was common between the viscera and body wall. This novel saponin contained four sugar residues. Silchenko, et al. [66] also reported an acetylated-sulphated tetraosides triterpene glycoside, Typicosides A_1_, isolated from the sea cucumber *Actinocucumis typica* (Family Cucumariidae, Order Dendrochirotida) with an identical *m*/*z* value (1221.5). However, the MS^2^ spectrum of the ions at *m*/*z* 1221.5 had a different fragmentation pattern from that recorded for Typicosides A_1_ even though they had the same *m*/*z* value which indicated the presence of a new saponin congener.

#### 2.5.2. Negative Ion Mode ESI-MS/MS

Negative ion mode MS/MS analyses were also performed on compounds under experimental conditions similar to those used for the positive ion mode. It is clear that fragmentation patterns produced in the negative ion mode MS/MS were different from those in the positive mode.

**Figure 10:**
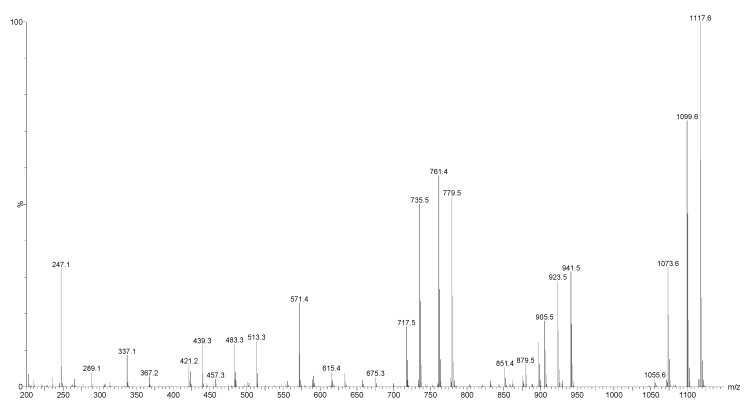
The ESI-MS/MS spectrum of desholothurin A in the negative ion mode.

As a typical example, the ESI-MS^2^ fingerprints of the ions at *m*/*z* 1117.6 [M − H]^−^ in the negative ion mode from fraction 110 is shown in Figure 10. These ions were observed at *m*/*z* 1141.5 [M + Na]^+^ in positive mode, which corresponded to desholothurin A (nobiliside 2a). This peak detected at *m*/*z* 1117 in the negative ion mode ESI-MS with molecular formula C_54_H_85_O_24_ [M − H]^−^, indicates the presence of one Na atom (sodium adduct in the positive mode) which means no sulphate group exists in this compound.

The mass discrepancy among these peaks and associated peaks in the positive ion mode were 24 u. For instance, the ions at *m*/*z* 337 and 483 corresponded to the ions at *m*/*z* 361 and 507 in the positive mode ESI-MS/MS, respectively. This analysis determined that the sugar compartment of this saponin comprised of four sugar residues. This analysis further validated our results.

### 2.6. Common Saponins between the Viscera and Body Wall

**Figure 11:**
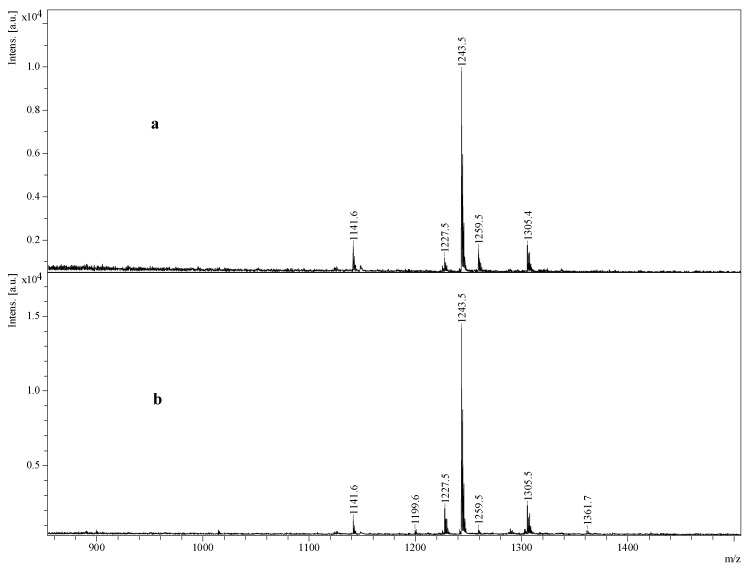
(+) MALDI spectra of butanolic saponin-enriched extract from viscera (**a**) and body wall (**b**) of *H. lessoni*.

Over 89 saponin congeners were found in the body wall, of which 54 saponin congeners have been reported previously. The comparison of saponins in the viscera and body wall of *H. lessoni*, showed that a large number (around 80 saponins) are shared between the body wall and the viscera as summarised in Table 1. Holothurin A was the major saponin in both body wall and viscera (Figure 11).

Even though the ions at *m*/*z* 1227.7 and 1229.5 were reported in both the body wall and viscera as major glycosides, our results revealed a higher abundance of these saponins in the body wall than in the viscera (Figure 11). The other compounds which gave a more intense signal in the body wall sample than the viscera sample were the ions at *m*/*z* 1291.5 and 1199.6, which corresponded to an unidentified saponin and arguside D, respectively. In contrast, the ions at *m*/*z* 1259.5 which corresponded to the sulphated isomeric compounds holothurins A_3_ and D [2,11,53,67], were more intense in the viscera as compared to the body wall.

Some saponin congeners including the ions detected at *m*/*z* 1123.5, 1125.5, 1141.5 1301.6, 1303.5, 1305.6, and 1307.5 were apparently found with similar intensities in both the body wall and viscera. These findings suggested that saponins were generated in both the body wall and viscera in various concentrations, which proposes a diverse function of saponins with different mechanisms of action. These data were in good agreement with the findings of Van Dyck et al. [55] who reported that saponins originated from different cells for different purposes.

The presence of a high percentage of saponins in both the organs indicated the main acceptable role for saponins: namely, the defensive function against different predators. However, the relative quantities of saponins were much higher in the viscera than in the body wall, which is in a good agreement with the literature. They might be responsible for unknown biological functions. In addition, there was a correlation between the content of saponins and their biological activities.

The saponin congeners identified in this species contained different key diagnostic peaks at 493, 507, 511, 523, 639, 657, and 673. For instance, the ion at *m*/*z* 1305 was a novel pentasaccharide triterpene glycoside which contained the key diagnostic peaks at *m*/*z* 507.2 and 639.6 corresponded to [MeGlc-Glc-Qui + Na]^+^ and [MeGlc-Glc-Qui-Xyl + Na]^+^, respectively. Further, it had an aglycone with a molecular weight of 486 Da.

A large number of identified saponins have been also reported in other species (Table 1). For instance, Kitagawa et al. [28] were the first to report the presence of 24-dehydroechinoside A or scabraside A in the cuvierian tubules of the sea cucumber *Actinopyga agassizi* Selenka. Han et al. [41] also found this compound in *H. scabra.* The structure of scabraside A was also described in the sea cucumber *H. scabra* using NMR and ESI techniques by Han et al. [38]. Fuscocinerosides A/B/C and pervicoside C were reported in the sea cucumber *Holothuria fuscocinerea* in which they differed in the lateral chains of their aglycones [37]. Fuscocineroside A is defined as an acetylated-sulphated tetraosides triterpene glycoside. Fuscocineroside C was also reported in the *H. scabra* [41]. Bondoc et al. [67] investigated saponin congeners in three species of Holothuriidae (*H. scabra* Jaeger 1833, *H. fuscocinerea* Jaeger 1833, and *H. impatiens* Forskal 1775). This group assigned peaks at *m*/*z* 1227 for fuscocinerosides B/C, 24-dehydroechinoside A or scabraside A and another isomer.

Chanley et al. [48] were the first to report the sugar components of holothurin A in the sea cucumber *A. agassizi* Selenka. Later, Kitagawa et al. [39] described the structure of holothurin A extracted from the cuvierian tubules of *H. leucospilota* using spectroscopy methods.

Holothurin A_3_, along with holothurin A_4_, were isolated primarily from the methanol extract of the sea cucumber *H. scabra* by Dang et al. [53]. This group indicated both holothurins A_3_ and A_4_ as sulphated tetrasaccharide triterpene glycosides, contacting sulXyl, Qui, Glc, and MeGlc at a ratio of 1:1:1:1, which were different in the lateral chain of their aglycone moieties.

#### 2.6.1. Unique Saponins in the Body Wall

The integrated HPCPC-MS analysis indicated the presence of 35 new and 54 reported saponins in the body wall. Of these, nine ions *m*/*z* 1069, 1103, 1189, 1459, 1461, 1463, 1489, 1535, and 1539, were found exclusively in the body wall as compared to the viscera. Most of them had high molecular weights ranging from *m*/*z* 1400 to 1600. This result indicated epidermal or adjacent epidermal states for these saponins (the outer body wall epithelium directing sea water) as Caulier, et al. [26] reported the ions at *m*/*z* 1463 in the seawater surrounding *H. lessoni.* Over 30 saponin congeners were found exclusively in the viscera compared to the body wall. These saponins could be involved in the regulation of the reproductive systems, acting as natural emulsifiers, and assisting the absorption of food in digestive organs or having defence mechanism [68,69].

Mass spectrometry analysis revealed that a saponin observed at *m*/*z* 1463.7, corresponding to holothurinosides H/H_1_, was localised exclusively in the body wall, probably in the epidermis. This observation was consistent with the findings proposed by Caulier, et al. [26] and Van Dyck, et al. [58] for the body wall of *H. lessoni* and *H. forskali*, respectively. Caulier et al. [26] reported the presence of this glycoside in the water surrounding the animal, which might have been released from the epidermis. Further, Van Dyck et al. [58] found this saponin congener localised in the epidermis of the body wall. Van Dyck, et al. [55] also indicated the presence of holothurinosides H/H_1_ in the cuvierian tubules of *H. forskali*, while cuvierian tubules were absent in *H. lessoni*. However, these ions (1463.7) were not detected in the viscera, indicating a particular localisation of this saponin, which might be generated by the further glycosylation of other saponins. Mitu et al. also reported the presence of three saponins in the conditioned water of *H. scabra* and stated they were generated by the body wall [16].

#### 2.6.2. Distribution of Saponin (Body Wall vs. Viscera)

Some of the identified saponins have been reported in several genera. For instance, the ion at *m*/*z* 1141 which corresponds to desholothurin A (synonymous with nobiliside 2a) or desholothurin A_1_ (synonymous with arguside E) was also reported in different species of sea cucumbers independently [28,29,30,32,40,55]. Desholothurin A was first detected in the sea cucumber *Actinopyga agassizi* Selenka [28].

Van Dyck and associates [58] examined the secretion of saponins in the challenged and non-stressed holothuroids. Holothurinoside G (*m*/*z* 1449) was the only saponin detected in the seawater surrounding non-stressed holothuroids, originating from the epidermis, while holothurinosides C (*m*/*z* 1125) and F (*m*/*z* 1433), and desholothurin A (*m*/*z* 1141) were secreted when the animals were stressed [58]. Further, they noted the presence of two saponins at *m*/*z* 1301 and 1317 (holothurinosides M and L, respectively) in water surroundings stressed holothuroids, which stemmed from an internal organ such as the respiratory trees rather than the epidermis. They concluded that the ions at *m*/*z* 1125, 1141, 1301, 1317, and 1433 were stress-specific saponins, which could play more vital defensive roles. However, these glycosides were noted in both the viscera and body wall of *H. lessoni*.

Van Dyck, et al. [58] reported saponins detected at *m*/*z* 1125 (holothurinosides C/C_1_), 1433 (holothurinosides F/F_1_), and 1449 (holothurinosides G/G_1_) present only in the epidermis, whereas saponins observed at *m*/*z* 1303 (Holothurinosides A/A_1_) were localised exclusively in the mesothelium, and saponins at *m*/*z* 1141 and 1287 were present in both epithelia of body wall of relaxed holothuroids. A saponin observed at *m*/*z* 1463 was mainly located in the epidermis, whereas one with an *m*/*z* value of 1479 showed no particular localisation.

A MALDI-MSI analysis of saponins from the cuvierian tubules showed that the prolonged stress situation modified Holothurinosides C/C_1_ (*m*/*z* 1125) to holothurinosides F/F_1_ and H/H_1_ (*m*/*z* 1433 and 1463, respectively), and desholothurins A/A_1_ (*m*/*z* 1141) to holothurinosides G/G_1_ and I/I_1_ (*m*/*z* 1449 and 1479, respectively) [55,58]. This occurred by the addition of a disaccharide; either Qui-Glc or MeGlc-Glc. This modification, addition of a disaccharide, increased the saponins hydrophobicity and membranolysis (i.e., more toxic) [70].

Ions at *m*/*z* 1287 and 1303 were localised in the mesothelial or near mesothelial (the inner body wall epithelium toward the coelomic cavity), while saponins at *m*/*z* 11xx and 14xx had an epidermal or adjacent epidermal state (the outer body wall epithelium) [55,58].

Van Dyck, et al. [55] also studied the cuvierian tubules of *H. forskali* in both relaxed and stressed conditions by MALDI-MSI to determine the localisation of saponins. Likewise in the body wall, they found eight major peaks at *m*/*z* 1125, 1141, 1287, 1303, 1433, 1449, 1463, and 1479 [55], and categorised them into three different groups, corresponding to the isomeric saponins, which corresponded to different physiological states. Further, they found saponin ions at *m*/*z* 1125 and 1141 in low concentrations exclusively in non-stimulated tissues. The second group, the most abundant saponins, noticed at *m*/*z* 1287 and 1303, was more localised in the connective tissue of both the stimulated and non-stimulated individuals’ tissues with the same concentration (expression level). They observed the third group of saponin ions at *m*/*z* 1433, 1449, 1463, and 1479 in the outer part of the connective tissue of the stressed specimen. They stated that the third group (*m*/*z* 14xx) were stress-specific and might originate from the first group (*m*/*z* 11xx) via glycosylation modifications. They also reported that different cell populations corresponded to generate different sets of saponins involving in a complex chemical defence mechanism [55]. For instances, holothurinosides A/A_1_ (*m*/*z* 1303) and E/E_1_ (*m*/*z* 1287) were produced by the vacuolar cells, while the other congeners generated by the neurosecretory-like cells. Recently, Popov and co-workers also investigated the distribution of saponin congeners in various organs of sea cucumber *Eupentacta fraudatrix* by LC-ESI QTOF-MS and stated the same metabolite profile for the whole body extract and the other individual analysed parts [71]. However, they reported the maximal content of the vast majority of detected compounds in the body wall as compared to other studied body components of sea cucumber. All the above findings support our data in which some saponin congeners were exclusively localised in the viscera or the body wall (present in only one type of organ), likely representing the specific and particular biological functions of these substances, while common congeners in the viscera and body wall might play the same role.

### 2.7. Bioactivity of Sea Cucumber Fractions and Saponins

#### 2.7.1. Antifungal and Antibacterial Activities of Purified Saponins

Sea cucumbers have been used as a traditional remedy to cure infectious diseases. Previous studies have shown that some triterpene glycosides isolated from sea cucumber species possess antifungal activity [72]. The antifungal activity of isobutanol-enriched saponin and HPCPC fractions from *H. lessoni* viscera and body wall were assessed against *Fusarium. pseudograminearum, Pythium. irregulare*, and *Rhizoctonia. solani.* Our results revealed that several tested saponin congeners (fractions) have strong antifungal activities against *F. pseudograminearum* and *R. solani*. The antifungal activities were defined by the diameter of the zones of inhibition

However, the examined triterpene glycosides had no effect on *P. irregulare*. Our data indicated that holothurian glycosides exhibit different activities against different fungal strains, which might be associated with the chemical composition and cellular structures of fungi.

Our result suggested that saponins having a linear sugar moiety, a sulphate group and/or an acetoxy group in their structures possess high antifungal activity. For instance, fractions that contained holothurin A and/or intercedenside A, which are sulphated compounds bearing a linear sugar residue, showed strong antifungal activity.

In contrast, the examined saponins had no inhibitory effect on the bacterial strain *S. aureus*, using the same concentration as used for the antifungal activity assay. This observation was consistent with the antibacterial findings of sea cucumber extracts reported by Mokhlesi et al. [73] and Kuznetsova et al. [74]. However, some studies reported antibacterial activity of sea cucumber saponins in crude extracts [75,76], which might be associated with other chemical classes rather than saponins.

#### 2.7.2. Anti-Oxidant Activity of Sea Cucumber Extracts

The antioxidant activities of different extracts (70% EtOH, MeOH, H_2_O, *i*-BuOH) of sea cucumber were evaluated by DPPH (2,2-Diphenyl-1-picrylhydrazyl) assay to determine their intrinsic antioxidant activity using α-tocopherol as the standard. Human immortalized keratinocytes (HaCat cells) were chosen as the target cells. Preliminary results indicated that sea cucumber extracts possess a high antioxidant activity in that the water extract and isobutanol fractions possess the highest antioxidant activity, which was consistent with the antioxidant findings reported by Husni et al. [77]. In summary, sea cucumber extracts tested in this experiment showed antioxidants activity comparable to other natural antioxidants.

## 3. Materials and Methods

### 3.1. Sea Cucumber Sample

Twenty sea cucumber samples of *Holothuria lessoni* were collected off Lizard Island (latitude 14°41′29.46″ S; longitude 145°26′23.33″ E), Queensland, Australia, in September 2010. The body wall was separated from the viscera (all internal organs) and kept separately in zip-lock plastic bags which were snap-frozen, then transferred to the laboratory and kept at −20 °C until use. The material and methods were the same as our previous publications [2,6,11], except for a small modification in the ESI-MS analysis as the samples were analysed in both the negative and positive ion modes.

### 3.2. Chemicals

All organic solvents were purchased from Merck (Darmstadt, Germany) except when the supplier was mentioned and was either of HPLC grade or the highest degree of purity. All aqueous solutions were prepared with ultrapure water generated by a Milli-Q system (18.2 MΩ, Millipore, Bedford, MA, USA).

### 3.3. Extraction and Purification Protocols

The saponins were extracted and purified as described previously [6,11], but by replacing the viscera with the body wall. The specimens were cut into small pieces, lyophilised and pulverised by a blonder and extracted with aqueous 70% EtOH (4 × 400 mL) on a shaker followed by filtration through Whatman filter paper (No.1, Whatman Ltd., Maidstone, UK) at room temperature overnight. The extract was concentrated under reduced pressure at 30 °C using a rotary evaporator (Büchi AG, Flawil, Switzerland) to remove the ethanol, and the residual sample was freeze dried. The dried extract (30 g) was re-dissolved in aq 90% MeOH (400 mL) and partitioned against 400 mL of n-hexane (*v*/*v*) twice. The water content of the hydromethanolic phase was then adjusted to 20% (*v*/*v*) and then to 40% (*v*/*v*) and the solutions partitioned against CH_2_Cl_2_ (450 mL) and CHCl_3_ (350 mL), respectively. The hydromethanolic phase was concentrated to dryness using a rotary evaporator and freeze drier. The dried powder was solubilized in 10 mL of MilliQ water (the aqueous extract) in readiness to undergo chromatographic purification.

### 3.4. Purification of the Extract

The aqueous extract was then subjected to Amberlite^®^ XAD-4 column chromatography (250 g XAD-4 resin 20–60 mesh; Sigma-Aldrich, MO, USA; 4 × 30 cm), washed extensively with water (1 L) to remove salts and impurities, and eluted sequentially with MeOH (450 mL), acetone (350 mL), and water (250 mL) [2,6,11]. The eluates were then concentrated, dried, and redissolved in 5 mL of MilliQ water. Finally, the aqueous extract was partitioned with 5 mL isobutanol (*v*/*v*). The isobutanolic saponin-enriched fraction was either stored for subsequent mass spectrometry analyses or concentrated to dryness and the components of the extract were further examined by HPCPC and RP-HPLC.

### 3.5. High-Performance Centrifugal Partition Chromatography (HPCPC or CPC)

The solvent system containing CHCl_3_:MeOH:H_2_O–0.1% HCO_2_H (7:13:8) was mixed vigorously in a separating funnel and allowed to reach hydrostatic equilibration [6,11]. Following the separation of the two-immiscible phase solvent systems, both phases were degassed using a sonicator-degasser (Soniclean Pty Ltd., Adelaide, SA, Australia). Then the rotor column of the dual mode HPCPC™, CPC240 (Ever Seiko Corporation, Tokyo, Japan) was filled with the lower stationary phase in the ascending mode at a flow rate of 5 mL min^−1^ by a Dual Pump model 214 (Tokyo, Japan), with a revolution speed of 300 rpm. The aqueous upper mobile phase was pumped in the ascending mode at a flow rate of 1.2 mL min^−1^ with a rotation speed of 900 rpm within 2 h. One hundred and forty milligrams of an isobutanol-enriched saponin mixture was then injected into the machine in the ascending mode. The injected sample was carried by the mobile phase. The chromatogram was developed for 3 h at 1.2 mL min^−1^ and 900 rpm using the Variable Wavelength UV-VIS Detector S-3702 (Soma optics, Ltd., Tokyo, Japan) and chart recorder (Ross Recorders, Model 202, Topac Inc., Cohasset, MA, USA). The fractions were collected in 3.5 mL tubes using a Fraction collector. At Fraction 73, the elution mode was switched to a descending mode and the lower organic phase was pumped at the same flow rate for 3 h to recover saponins. The profile of fractions was also monitored by TLC. Monitoring of the fractions was necessary as most of the saponins could not be detected by UV due to the lack of a chromophore structure. Fractions were concentrated with nitrogen gas.

### 3.6. Thin Layer Chromatography (TLC)

Ten microliters of all fractions were applied on silica gel 60 F_254_ aluminium sheets (Merck # 1.05554.0001, Darmstadt, Germany) and developed with the lower phase of a CHCl_3_:MeOH:H_2_O (7:13:8 *v*/*v*/*v*) biphasic solvent system. The profile of separated compounds on the TLC plate was visualized by UV light, and by spraying with a 15% sulfuric acid in EtOH solution and heating for 10 min at 110 °C until maroon-dark purple spots developed.

### 3.7. Mass Spectrometry

The isobutanol saponin-enriched fractions and the resultant HPCPC purified polar samples were further analyzed by MALDI and ESI MS to elucidate and characterize the molecular structures of compounds. Mass spectrometry analyses combined with the existing literature led to the discovery of many known and new glycosides.

#### 3.7.1. MALDI

MALDI mass spectra were acquired using a Bruker Autoflex III Smartbeam (Bruker Daltonik, Bremen, Germany). All MALDI MS equipment, software, and consumables were from Bruker Daltonics. The laser (355 nm) had a repetition rate of 200 Hz and operated in the positive reflectron ion mode for MS data over the mass range of 400 to 2200 Da under the control of the Flexcontrol and FlexAnalysis software (V3.3 build 108) (Bruker Daltonik, Bremen, Germany). External calibration was conducted using the sodium-attached ions from a Polyethylene Glycol (PEG) of average molecular weight 1000. MS spectra were processed in FlexAnalysis (V3.3, Bruker Daltonik, Bremen, Germany). MALDI MS^2^ spectra were acquired in the LIFT mode of the Bruker Autoflex III with the aid of CID. The mass-selected ions were subjected to collision against argon in the collision cell to be fragmented, affording intense product ion signals. For MALDI, a laser was used to provide both good signal levels and mass resolution with the laser energy for MS^2^ analysis being generally 25% higher than for MS analysis.

The samples were loaded onto a MALDI stainless steel MPT Anchorchip TM 600/384 target plate. Alpha-cyano-4-hydroxycinnamic acid (CHCA) in acetone/iso-propanol in a ratio of 2:1 (15 mg mL^−1^) was used as a matrix to produce gas-phase ions. The matrix solution (1 μL) was spotted on the MALDI target plate and air-dried. Subsequently, 1 μL of sample was added to the matrix crystals and air-dried [2,6,11]. Finally, 1 μL of a NaI (Sigma-Aldrich # 383112, St Louis, MI, USA) solution (2 mg/mL in acetonitrile) was applied to the sample spots. The samples were mixed on the probe surface and dried prior to analysis. The dried samples were then introduced to MALDI for analysis.

Typically, the analysis of saponins by MALDI and ESI in the positive ion mode yields sodium adducts ions [M + Na]^+^, however, protonated [M + H]^+^ and potassium-cationized [M + K]^+^ saponin ions are also observed.

#### 3.7.2. ESI MS

The ESI mass spectra were attained with a Waters Synapt HDMS (Waters, Manchester, UK). Mass spectra were acquired in both the positive and negative ion modes with a capillary voltage of 3.0 kV and a sampling cone voltage of 60 V.

The other conditions were as follows: extraction cone voltage, 4.0 V; ion source temperature, 80 °C; desolvation temperature, 350 °C; desolvation gas flow rate, 500 L·h^−1^ [2,11]. Data acquisition was performed using a Waters MassLynx (V4.1, Waters Corporation, Milford, CT, USA). Positive ion mass spectra were obtained in the V resolution mode over a mass range of 600–1600 *m*/*z* using the continuum mode acquisition. Mass calibration was performed by infusing a sodium iodide solution (2 μg/μL, 1:1 (*v*/*v*) water:isopropanol). An accurate mass analysis was conducted in the positive ion mode, a lock mass signal from the sodium attached molecular ion of Raffinose (1 ng/μL in 50% aq acetonitrile, *m*/*z* 527.1588) was used through the LockSpray source of the Synapt instrument.

MS^2^ spectra were acquired by mass selection of the ions of interest using the quadrupole fragmentation in the trap cell where argon was used as collision gas. The typical collision energy (Trap) was 50.0 eV. Samples were infused at a flow rate of 5 µL/min; if the dilution of the sample was required then acetonitrile was used.

### 3.8. Antifungal Activity Assay (Plug Type Diffusion Assay)

The antifungal activities of the isobutanol-saponin enriched and HPCPC fractions (pure saponins) were tested against three strains including *Fusarium pseudograminearum, Pythium irregulare*, and *Rhizoctonia solani* using a modified disc diffusion agar assay [78]. The test fungi were grown on an HPDA medium for 7 days, and a plug of the radial growth of each fungus was cut (0.5 × 0.5 cm cubes). The cubes were then placed onto the centre of a new HPDA plate and incubated at 27 °C for 24 h, or until the fungal growth surrounding the cube extended to a 1.5 cm diameter. At this stage, 40 µL of the samples (in methanol, in duplicate) were spotted onto standard paper discs and air-dried. The six discs were then placed onto the fungal growth plates about 1.5 cm from the edge and pressed into the agar using sterile tweezers. The plates were then re-incubated at 27 °C and checked for inhibition zones every 24 h for four days. The negative controls were methanol and plates of each fungus culture with tested samples, while Benomyl ^®^ (Sigma-Aldrich, Castle Hill, Australia; 50 µg/mL) was used as a positive control.

### 3.9. Antibacterial Activity Assay

The antibacterial activity of saponin extracts was examined against Gram-positive bacterium *Staphylococcus aureus* using a typical agar diffusion assay. An antibiotic assay medium No.1 (AAM) was used for the antibacterial activity assay modified from Almuzara, et al. [79] and Wikler [80]. The test culture was grown in tryptone soy broth (TSB) and incubated at 37 °C for 18–22 h. The growth of the culture was evaluated by measuring the optical density (OD) using a Shimadzu UV-160A spectrophotometer at 600 nm (OD600 nm), and the OD was adjusted to 0.2. The AAM was seeded with the culture (1% *v*/*v*) and dispensed into 9-cm petri dish plates at 25 mL/plate, and cut using a cork borer to make 10 wells (6 mm). Each well was then filled with 40 µL of samples (in methanol) and the plates were incubated at 37 °C for 18–24 h. Vancomycin (0.25 µg/mL) was used as a positive control.

## 4. Conclusions

Sea cucumbers have been utilised as traditional folk remedies to treat various ailments by traditional practitioners. Sea cucumbers are a rich source of novel and bioactive metabolites. They are commercially important and contain various potent substances that can be used as a health care product in the markets. Among them, saponins are the most important and prime secondary metabolites reported in sea cucumbers. Likewise, the viscera, a highly diverse range of saponin congeners was identified in the body wall. This vast diversity could be associated with the different roles of saponins in sea cucumbers including kairomones; as chemical communicates to attract symbionts, chemical defence mechanism; the most acceptable biological functions for these bioactive compounds, or aposematic signal; threatening potential predators of the unpalatability food. Saponins are considered as a defence mechanism in which they are deleterious for most organisms, based on either adhesive defence or toxic mechanisms. The presence of a large number of the common saponins in both organs demonstrates their multifunctionality, representing the different internal and external biological roles of these metabolites.

Profiling of *H. lessoni* was conducted by MALDI and ESI-MS. The integration of HPCPC, MALDI-MS, ESI-MS, and tandem mass spectrometry proved to be a very efficient combination for structure elucidation of saponin congeners. The interpretation of fragmentation patterns of MS/MS spectra of triterpene glycosides allowed for the characterisation of the chemical structure of saponins. Accordingly, this analysis revealed the presence of 89 saponins. Knowledge of the chemical structure of saponins is critical for better understating of their structural/ activity relationships as well as the biosynthesis and biological roles of these compounds.

This study highlighted the diversity of saponin congeners in the viscera and body wall. This species produced a diverse range of saponin congeners, many of which were common between the body wall and the viscera. The results also revealed that some saponins are organ-specific. In other words, the different organs are characterised by different saponin congeners or specific saponin contents. Some of them were specific to either the viscera or the body wall. Further, the MS analyses also indicated that this species produced a mixture of common and unique saponin types. This specific localisation might be attributed to a particular function of these congeners, which will require further studies. The viscera had the highest number of specific congeners, which interestingly the majority belonged to non-sulphated triterpene glycosides. The role of viscera-specific triterpene glycosides may associate with regulating the reproduction of sea cucumbers, which is in a very good corroboration of the internal biological function of saponins. This indicated that the identity of saponins generated by sea cucumbers are different from species to species.

The most abundant ions observed under positive ion conditions were mainly sulphated compounds, which were common between the viscera and the body wall. This study suggested that saponins were synthesised in both the viscera and body wall, but further studies are warranted to investigate the biosynthesis of these secondary metabolites to discover which cells are in charge of producing saponins.

Saponin extracts are complex mixtures and, as such, the isolation and purification of these natural compounds are tedious, labour-consuming, and multistage due to their low content and a large number of saponin isomers. However, the identification of a large number of saponin congeners was not only due to the availability, development, and implementation of cutting-edge analytical equipment such as mass spectrometry and HPCPC based-procedures, but was also due to the presence of isomeric congeners in the experimental extract.

Many analytical methods have been used to purify, determine and elucidate the structure of saponin congeners in marine animals. As such a high diversity of saponin congeners were reported in this organism. In the current work, a large number of saponin congeners were detected for the first time using both the positive and negative modes of mass spectrometry. The structure of four novel acetylated saponins, namely lessoniosides H, I, J, and K were characterized.

In conclusion, our data revealed that there were differences in the distribution of saponins between the body wall and viscera, and showed a higher number of saponins for the viscera than the body wall, and highlighted some saponin congeners were found exclusively in the viscera.

However, some highly glycosylated saponins, such as ions at *m*/*z* 1461 and 1463, were found only in the body wall. In fact, having large sugar moieties increase the water solubility of these molecules. The examined saponins indicted a strong antifungal and antioxidant activities. This study revealed that sea cucumbers produce a wide spectrum of saponins with potential applications as valuable functional food or nutraceuticals as well as functional ingredients for cosmeceutical, medicinal, pharmaceutical products to improve human health.

## Figures and Tables

**Table 1:** The summary of saponins identified from the body wall of *H. lessoni* by MALDI- and ESI-MS^2^. This table illustrates the 35 novel identified compounds (N) along with the 54 known compounds (P). This table also shows some identical saponins, which have been given different names by different researchers in which they might be isomeric congeners. Besides, it addresses the presence of specific saponins in the viscera or the body wall.

[M + Na]^+^ *m*/*z*	MW	Formula	Compound Name	Body Wall	Viscera	Novel (N)/Published (P)	References
889.4	866	C_41_H_63_NaO_16_S	Holothurin B_3_	Yes	Yes	P	[19]
		C_42_H_67_NaO_15_S	Unidentified	Yes	Yes	N	−
905.4	882	C_41_H_63_NaO_17_S	Holothurin B_4_	Yes	Yes	P	[2,19]
			Holothurin B	Yes	Yes	P	[20,21]
			Nobiliside B	Yes	Yes	P	[22]
907.4	884	C_41_H_65_NaO_17_S	Holothurin B_2_	No	Yes	P	[19]
			Leucospilotaside B	No	Yes	P	[23]
911.6	888	C_45_H_92_O_16_	Unidentified	Yes	Yes	N	−
917.4	994	C_44_H_71_NaO_15_S	Unidentified	No	Yes	N	−
921.4	898	C_41_H_63_NaO_18_S	Leucospilotaside A	No	Yes	P	[24]
1034.1	1011	a *	Unidentified	Yes	Yes	N	−
1065.5	1042	C_48_H_82_O_24_	Unidentified	No	Yes	N	−
1069.5	1046	C_52_H_86_O_21_	Unidentified	Yes	No	N	-
1071.5	1048	C_47_H_93_NaO_21_S	Unidentified	Yes	Yes	N	[2,11]
1079.5	1056	C_53_H_84_O_21_	Unidentified	Yes	Yes	N	-
1083.3	1060	C_58_H_64_O_25_	Unidentified	No	Yes	N	[2,11]
1085.5	1062	C_53_H_90_O_21_	Unidentified	No	Yes	N	-
1087.5	1064	C_52_H_88_O_22_ C_47_H_93_NaO_22_S	Unidentified	Yes	Yes	N	[2,11]
1101.6	1078	C_52_H_86_O_23_	Unidentified	Yes	Yes	N	-
1103.5	1080	C_52_H_88_O_23_	Unidentified	Yes	No	N	-
1107.7	1084	C_54_H_84_O_22_	Unidentified	Yes	Yes	N	-
1109.5	1086	C_54_H_86_O_22_	DS-pervicoside B	Yes	Yes	P	[25]
1111.5	1088	C_54_H_88_O_22_	Bivitoside B	Yes	Yes	P	[26,27]
1121.5	1098	C_54_H_82_O_23_	Unidentified	No	Yes	N	-
1123.5	1100	C_54_H_84_O_23_	Unidentified	Yes	Yes	N	[2,11]
1125.5	1102	C_54_H_86_O_23_	Holothurinosides C/C_1_	Yes	Yes	P	[28,29]
1127.6	1104	C_53_H_84_O_24_C_54_H_88_O_23_	Holothurinosides X/Y/Z	Yes	Yes	P	[2,11]
1139.5	1116	C_54_H_84_O_24_	Unidentified	No	Yes	N	-
1141.5	1118	C_54_H_86_O_24_	Desholothurin A (Nobiliside 2a), Desholothurin A_1_ (Arguside E)	Yes	Yes	P	[2,28,29,30,31,32,33]
1149.2	1126	a *	Holothurinoside T	No	Yes	P	−
1157.5	1134	C_54_H_86_O_25_	Holothurinoside J_1_ Unidentified	Yes	Yes	P N	[2,11,34]
1163.5	1140	C_54_H_92_O_25_	Unidentified	Yes	Yes	N	-
1167.8	1144	C_56_H_88_O_24_	Arguside A	No	Yes	P	[35]
1173.5	1150	C_57_ H_82_O_24_	Unidentified	Yes	Yes	N	-
1179.5	1156	C_57_H_88_O_24_C_54_H_85_NaO_23_S	Unidentified	Yes	Yes	N	-
1181.4	1158	C_57_H_90_O_24_	Unidentified	No	Yes	N	-
1189.5	1166	C_59_H_97_O_24_	Unidentified	Yes	No	N	-
1193.5	1170	C_54_H_83_NaO_24_S	Unidentified	Yes	Yes	N	[2,11]
1197.5	1174	C_54_H_87_NaO_24_S	Unidentified	Yes	Yes	N	-
1199.5	1176	C_54_H_64_O_29_C_56_H_88_O_26_	Unidentified Arguside D	Yes	Yes	N P	[2,31]
1205.5	1182	C_57_H_82_O_26_ C_55_H_83_NaO_24_S	Unidentified	Yes	Yes	N	-
1207.5	1184	C_55_H_83_NaO_24_S	Unidentified	Yes	Yes	N	-
1211.5	1188	C_54_H_85_NaO_25_S	Unidentified	Yes	Yes	N	-
1221.5	1198	C_56_H_78_O_28_C_55_H_83_NaO_25_S	Unidentified Intercedenside A	Yes	Yes	N P	[2,36]
1223.5	1200	C_55_H_85_NaO_25_S	Unidentified	No	Yes	N	-
1225.5	1202	C_54_H_83_NaO_26_S	Unidentified	No	Yes	N	−
1227.5	1204	C_54_H_85_NaO_26_S	Fuscocinerosides B/C, Scabraside A or 24–dehydroechinoside A, Unidentified	Yes	Yes	P	[11,28,37,38,39,40,41,42]
1229.5	1206	C_54_H_87_NaO_26_S	Holothurin A_2_, Echinoside A Pervicoside B	Yes	Yes	P	[20,26,40,43,44,45,46]
1237.5	1214	C_56_H_78_O_29_C_55_H_83_NaO_26_S	Unidentified	Yes	Yes	N	-
1243.5	1220	C_54_H_85_NaO_27_S	Holothurin A Scabraside B 17-Hydroxy fuscocineroside B, 25-Hydroxy fuscocinerosiden B	Yes	Yes	P	[19,20,33,38,39,46,47,48,49,50,51,52]
1245.5	1222	C_54_H_87_NaO_27_S	Holothurin A_1_ Holothurin A_4_ Scabraside D	No	Yes	P	[40,41,53]
1259.5	1236	C_54_H_85_NaO_28_S	Holothurin A_3_ Holothurin D	Yes	Yes	P P	[2,11,53]
1261.5	1238	C_54_H_87_NaO_28_S	Unidentified	No	Yes	N	−
1265.5	1242	C_56_H_83_NaO_27_S	Unidentified	Yes	Yes	N	[2]
1269.5	1246	C_60_H_94_O_27_	Cousteside G	No	Yes	P	[32]
1271.6	1248	C_60_H_96_O_27_	Impatienside B Cousteside H	Yes	Yes	P	[32,54]
1273.6	1250	C_60_H_98_O_27_	Cousteside J	Yes	Yes	P	[2,32]
1281.4	1258	C_54_H_87_NaO_29_S	Unidentified	No	Yes	N	-
1283.4	1260	C_54_H_89_NaO_29_S C_61_H_96_O_27_	Unidentified	No	Yes	N	-
1285.6	1262	C_56_H_87_NaO_28_S	Fuscocineroside A	Yes	Yes	P	[37]
1287.6	1264	C_60_H_96_O_28_	Holothurinoside E,	Yes	Yes	P	[30,55]
			Holothurinoside E_1_	Yes	Yes	P	[30,55]
			Holothurinoside O	Yes	Yes	P	[2,11]
			Holothurinoside P	Yes	Yes	P	[2,11]
			17-dehydroxy holothurinoside A	Yes	Yes	P	[32,56]
			Cousteside E	Yes	Yes	P	[32]
			Cousteside F	Yes	Yes	P	[32]
		C_56_H_89_NaO_28_S	22-acetoxy-echinoside A	Yes	Yes	P	[57]
1289.6	1266	C_60_H_98_O_28_	Griseaside A Cousteside I	Yes Yes	Yes Yes	P P	[56] [32]
1301.6	1278	C_61_H_98_O_28_C_60_H_94_O_29_	Holothurinoside M Unidentified	Yes	Yes	P N	[11,58]
1303.6	1280	C_60_H_96_O_29_	Holothurinoside A	Yes	Yes	P	[29,30]
			Holothurinoside A_1_	Yes	Yes	P	[29,30]
			Holothurinoside Q	Yes	Yes	P	[2,11]
			Holothurinoside S	Yes	Yes	P	[2,11]
			Holothurinoside R	Yes	Yes	P	[2,11]
			Holothurinoside R_1_	Yes	Yes	P	[2,11]
			Cousteside C	Yes	Yes	P	[32]
1305.6	1282	C_60_H_98_O_29_	Unidentified	Yes	Yes	N	[2]
1307.6	1284	C_60_H_100_O_29_	Unidentified	Yes	Yes	N	[2]
1317.6	1294	C_61_H_98_O_29_	Unidentified Holothurinoside L	Yes	Yes	N P	[2,11,26]
1319.5	1296	C_60_H_96_O_30_	Unidentified	Yes	Yes	N	-
1329.7	1306	C_62_H_98_O_29_	Arguside F	No	Yes	P	[54]
1335.3	1312	C_60_H_96_O_31_	Unidentified	Yes	Yes	N	[2]
1349.8	1326	C_61_H_98_O_31_	Unidentified	No	Yes	N	-
1356.4	1333	a *	Unidentified	No	Yes	N	−
1361.7	1338	C_63_H_102_O_30_	Unidentified	Yes	Yes	N	-
1377.3	1354	C_63_H_102_O_31_	Unidentified	No	Yes	N	-
1409.4	1386	C_61_H_78_O_36_	Unidentified	Yes	Yes	N	[2]
1411.7	1388	C_62_H_116_O_33_	Unidentified	No	Yes	N	−
1415.7	1392	C_66_H_104_O_31_	Unidentified	No	Yes	N	-
1417.7	1394	C_66_H_106_O_31_	Unidentified	Yes	Yes	N	-
1419.7	1396	C_66_H_108_O_31_	Unidentified	Yes	Yes	N	[2]
1431.4	1408	C_66_H_104_O_32_	Unidentified	No	Yes	N	-
1435.7	1412	C_66_H_108_O_32_	Unidentified	Yes	Yes	N	[2]
1447.7	1424	C_67_H_108_O_32_	Unidentified Impatienside A Marmoratoside A	Yes	Yes	N P	- [59]
1449.7	1426	C_67_H_110_O_32_	Bivittoside D	No	Yes	P	[27]
1453.6	1430	C_66_H_94_O_34_	Unidentified	Yes	Yes	N	-
1459.7	1436	C_68_H_108_O_32_	Unidentified	Yes	No	N	-
1461.7	1438	C_68_H_110_O_32_	Unidentified	Yes	No	N	-
1463.7	1440	C_67_H_108_O_33_	Holothurinosides H/H_1_ Holothurin C Cousteside A 17α-hydroxy impatienside A Marmoratoside B	Yes	No	P	[26,55] [32] [59]
1465.7	1442	C_67_H_110_O_33_	Argusides B/C	No	Yes	P	[60]
1475.7	1452	C_68_H_108_O_33_C_65_H_112_O_35_	Unidentified	Yes	Yes	N	[11]
1477.7	1454	C_68_H_110_O_33_C_65_H_114_O_35_	Lessoniosides A/B/C/D/E Unidentified	Yes	Yes	P	[6]
1479.7	1456	C_67_H_108_O_34_	Holothurinosides I/I_1_	No	Yes	P	[55]
1481.7	1458	C_66_H_106_O_35_C_67_H_110_O_34_	Unidentified	Yes	Yes	N	[2]
1489.7	1466	C_68_H_106_O_34_	Unidentified	Yes	No	N	-
1491.5	1468	C_68_H_108_O_34_	Unidentified	No	Yes	N	-
1493.7	1470	C_68_H_110_O_34_C_65_H_114_O_36_	Unidentified	No	Yes	N	−
1495.7	1472	C_67_H_108_O_35_	Holothurinoside K_1_ Unidentified	No	Yes	P N	[34] −
1507.7	1484	C_69_H_112_O_34_	25-acetoxy bivittoside D	Yes	Yes	P	[59]
1521.7	1498	C_69_H_110_O_35_	Unidentified	Yes	Yes	N	-
1535.7	1412	C_69_H_108_O_36_	Unidentified	Yes	No	N	-
1539.7	1416	C_69_H_112_O_36_	Unidentified	Yes	No	N	-
1591.7	1568	C_66_H_120_O_41_	Unidentified	No	Yes	N	−

a * The composition was not measured through the ESI analysis.
